# Atypical Postpartum Psychosis and Neuropsychiatric Manifestations Coexisting With Global Cortical Atrophy

**DOI:** 10.7759/cureus.98177

**Published:** 2025-11-30

**Authors:** Brandon J Hegele, Stephen A Zorc, Ugoma Onubogu, Benjamin Oakes, Peter Averkiou

**Affiliations:** 1 College of Medicine, Florida Atlantic University Charles E. Schmidt College of Medicine, Boca Raton, USA; 2 College of Medicine, University of Central Florida College of Medicine, Orlando, USA

**Keywords:** anoxic brain injury, delusions, diffuse cortical atrophy, hallucinations, hypoxic-ischemic brain injury, postpartum hemorrhage, postpartum psychosis, psychosis, schizophrenia

## Abstract

The neuropsychiatric sequelae resulting from postpartum hemorrhage (PPH)-induced hypoxic-ischemic brain injury (HIBI) are sparsely documented, presenting a complex diagnostic and therapeutic challenge for clinicians. We report the case of a 47-year-old woman who experienced PPH 13 years before her current presentation. Her PPH resolved following a partial hysterectomy and multiple transfusions, resulting in a coma from which she awoke with vivid hallucinations and altered memory. These symptoms initially improved, although she never regained baseline function and continued to experience cognitive distortion and hallucinations. She now presents 13 years later with an acute exacerbation of her symptoms after missing a dose of her long-acting injectable antipsychotic and continues to experience entrenched delusions involving false children, family, and occupational identities. Computed tomography of her brain at this presentation revealed global cortical atrophy disproportionately severe for her age, suggesting an organic cause of her symptoms. Her insight was poor, and she was unable to recognize the sequelae of her condition. During admission, her mental status rapidly progressed from cooperative to agitated as the health care team communicated their findings, which she deemed to be falsification and lies. This case underscores a unique neuropsychiatric manifestation secondary to severe PPH and HIBI with subsequent postpartum psychosis (PPP). HIBI can trigger or exacerbate psychosis and cognitive distortions through mechanisms including neuroinflammation in the acute phase, or structural brain alterations and neurotransmitter imbalances in the chronic phase. These mechanisms likely contribute to her mood disturbances, auditory and visual hallucinations, and false memories. Similar HIBIs of varying etiologies have been linked to diffuse cortical atrophy, as observed in this patient. Moreover, PPP is notable for its rapid onset and intensity, yet few cases detail progression to chronic neuropsychiatric symptoms without full recovery. This case represents a rare, chronic neuropsychiatric presentation following PPH and subsequent HIBI, emphasizing the need for vigilance in managing emerging psychiatric conditions after obstetric hemorrhage. Further research into neuropsychiatric outcomes after complicated PPH and hypoxic events is warranted to improve diagnostic and therapeutic strategies.

## Introduction

Postpartum psychosis (PPP) is a rare and severe psychiatric emergency with an estimated incidence of one to two per 1,000 deliveries [1]. It is characterized by rapid-onset psychotic symptoms, typically emerging within the first two weeks postpartum and often including delusions, hallucinations, mood instability, and cognitive disorganization [2-4]. While frequently associated with underlying bipolar spectrum disorders, PPP can also occur in women without a prior psychiatric history [2,4]. Although most cases are time-limited and resolve with appropriate treatment, a subset of patients may experience persistent neuropsychiatric impairment, especially in the context of obstetric complications [5-7].

Postpartum hemorrhage (PPH) is a leading cause of maternal morbidity and mortality. PPH can result in significant hypovolemic shock and, in rare cases, hypoxic-ischemic brain injury (HIBI) [8]. The intersection of severe PPH, prolonged cerebral hypoperfusion, and psychiatric decompensation presents a diagnostic and therapeutic challenge that is rarely documented in the literature [8,9]. The long-term neuropsychiatric sequelae of HIBI following obstetric complications remain underexplored, particularly regarding their influence on persistent psychosis, memory dysfunction, and dissociative phenomena [8,9].

This case highlights the potential for chronic psychiatric syndromes to emerge in the aftermath of obstetric emergencies and underscores the critical need for interdisciplinary collaboration among psychiatrists, obstetricians, and neurologists in both acute and long-term management [5,6,10,11]. It also calls attention to a possible novel variant of PPP with enduring features, shaped not solely by psychiatric vulnerability but also by structural brain changes associated with obstetric complications and hemorrhage [10,11].

## Case presentation

A 47-year-old woman with a reported history of schizophrenia and bipolar disorder presented to the emergency department (ED) under involuntary psychiatric hold for concerns of self-neglect and harm to others. The patient’s parents initiated the hold after noting a marked exacerbation of psychotic symptoms, including disorganized behavior, persecutory delusions, and grandiose delusions. The inciting incident involved the patient attempting to purchase an expensive out-of-town taxi service to a location two hours away to reunite with her husband and children. When her father attempted to intervene by removing her phone, she became physically aggressive and tried to bite him. Collateral history revealed that the patient had missed a scheduled administration of long-acting injectable (LAI) antipsychotic medication because her physician was on vacation. Her active medications at presentation were lithium extended release 600 mg daily for bipolar disorder, LAI fluphenazine 25 mg every two weeks for psychosis, trihexyphenidyl 2 mg twice daily for extrapyramidal side effects of antipsychotics, clonazepam 1 mg nightly for restlessness, and levothyroxine 50 mcg daily for hypothyroidism.

In the ED, the patient appeared calm but mildly anxious. On introduction, she was well poised and well spoken. She cohesively recalled the events preceding her arrival and denied any psychiatric history. She recalled her attempt to purchase a taxi to see her husband and four children, justifying her episode of aggression with the wrongdoings of her parents. The patient denied any suicidal or homicidal ideation and denied auditory or visual hallucinations. She denied any substance use. Her speech was fluent and circumferential; affect was flat, and she described her mood as “okay.” Her insight was poor, and she was unable to appreciate her condition at the time. Her judgment and reasoning appeared intact initially, although further questioning revealed a shifting story that did not fit her initial claims. It became clear through the interview that her delusions dictated her reality and influenced her decision-making ability. She was reluctant to engage with the treatment team but remained cooperative. She exhibited occasional accusatory remarks and minor oral automatisms, such as lip smacking. It was not until receiving collateral information that it became evident that confabulation and delusions dictated her narrative. 

Collateral information provided by family members contradicted much of the seemingly cohesive story the patient had presented. Her parents reported a history of three late-term fetal demises, with the final miscarriage complicated by PPH and coma, after which psychiatric disturbances began. Following her last fetal demise 14 years ago, she was hospitalized and subsequently discharged home. Two days after discharge, her mother reported that the patient appeared very pale and lost consciousness upon standing in their home. She was then brought back to the hospital, where she was found to have a PPH and required multiple blood transfusions.

She remained in a medically induced coma for two days in the ICU. Upon regaining consciousness, she began experiencing auditory and visual hallucinations as well as amnesia. She described seeing snakes on the walls of her hospital room and was unaware of her recent fetal loss. No formal neuropsychiatric evaluation was conducted at that time.

In the aftermath of her PPH, the patient became divorced and moved in with her parents. She began receiving Social Security Disability Insurance during the immediate period after her PPH. They confirmed that she has no living children. Over the past 12 years, she has been dependent on her parents for activities of daily living, such as financial and medication management. Four years prior to her current presentation (eight years after the PPH), she experienced an episode of agitation that resulted in an ED visit. She was subsequently admitted to a psychiatric facility, where she was diagnosed with bipolar I disorder and schizophrenia. She was started on antipsychotic medication and followed up with an outpatient psychiatrist thereafter.

While living with her parents, the patient would routinely apply makeup and prepare for work each day, but would then remain in her bedroom for most of the day, speaking to people who were not present. She often became distressed about deadlines and meetings related to her previous line of work.

**Figure 1:**
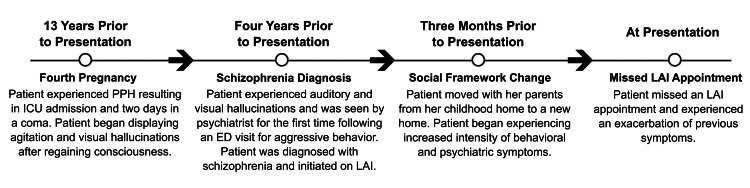
Representative timeline of clinically relevant patient events, symptomatology, and medical history before her most recent hospital presentation. PPH: postpartum hemorrhage; ICU: intensive care unit; ED: emergency department; LAI: long-acting injectable

Three months prior to her current presentation, the patient and her family moved from their long-term home, after which her symptoms worsened. Figure 1 depicts a representative timeline of significant events.

When questioned about her parents’ statements, the patient claimed that they were lying and expressed confusion about their motives for deceiving the treatment team. She maintained that she and her children still lived in their previous home, endorsed working as a federal agent, and claimed to hold a PhD, all of which were false. When asked to name her children, she provided four names in rapid succession, each sharing the same first syllable and terminal rhyme.

Collateral information provided insight into the years prior to the patient's PPH. While the patient did not carry any psychiatric diagnoses prior to her schizophrenia and bipolar I diagnosis four years before her current presentation, her parents described the onset of behavioral disturbances earlier in her life. During her late high school years, she was described as having episodes of behavioral outbursts that co-occurred with interpersonal stressors such as breakups. Her parents reported two episodes of attempted suicide in which she cut her wrists following breakups with boyfriends. Despite this, she was successful in school and work, and her condition did not significantly impact her ability to live a normal life. Her parents denied any delusional thought content, agitation, or hallucinations prior to her PPH. Her medical and surgical history prior to her PPH was unremarkable, besides her aforementioned obstetric complications.

Laboratory tests were conducted to assess for causes of altered mental status, metabolic syndrome, and endocrine dysfunction. Laboratory examination of electrolytes, thyroid function tests, and lipid panel identified no gross abnormalities (Table 1). Assessment of serum lithium revealed a concentration below the therapeutic range at 0.28 millimole per liter. Serum ethanol was below detection at <10 mg/dL. An 11-panel urine drug screen was grossly negative. A qualitative urine pregnancy test was negative.

**Table 1:** Results of laboratory testing.

Laboratory Test	Result	Reference Range
Sodium	136	136 to 145 mmol/L
Potassium	3.8	3.6-5.2 mmol/L
Chloride	103	96-106 mmol/L
Bicarbonate	27	23-29 mEq/L
Glucose	132	72-99 mg/dL
Blood Urea Nitrogen	10	2.1 to 8.5 mmol/L
Creatinine	1	0.59-1.04 mol/L
Estimated Glomerular Filtration	70	90-100 ml/min
Alkaline Phosphatase	77	30-120 U/L
Alanine Transaminase	27	8-33 U/L
Aspartate Transaminase	17	4-36 U/L
Calcium	9.6	8.6-10.3 mmol/L
Anion Gap	6	4-12 mEq/L
Serum Osmolality	283	275-295 mOsm/kg
Thyroid-Stimulating Hormone	2.76	1.7-2.2 mg/dL
Free T4 (Thyroxine)	0.71	2.5-4.5 mg/dL
Cholesterol	152	<200 mg/dL
High-Density Lipoprotein	41	>50 mg/dL
Low-Density Lipoprotein	86	<100 mg/dL
Triglycerides	124	<150 mg/dL
White Blood Cell Count	10.6	4.5-11x109/L
Hemoglobin	12.6	12 to 15.5 g/L
Platelet Count	364	150-400x103/µL
Hematocrit	39.2	36-44%

**Figure 2:**
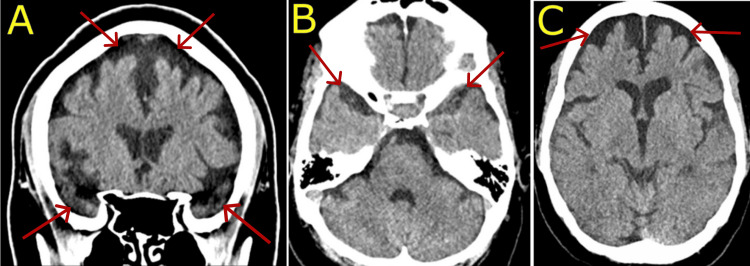
A) Coronal CT image showing diffuse cortical atrophy (red arrows, above) and loss of temporal lobe density (red arrows, below). B) Axial CT image showing loss of temporal lobe density (red arrows). C) Axial CT image showing diffuse cortical atrophy (red arrows).

Neuroimaging was performed to assess organic contributions to her psychiatric symptoms. A non-contrast head computed tomography (CT) scan revealed global cortical atrophy disproportionately severe for her age and loss of temporal lobe density (Figure 2). Neurology was consulted to evaluate the possible sequelae of a prior hypoperfusion injury related to PPH. Although magnetic resonance imaging (MRI) was ordered for further evaluation, the patient refused to undergo further imaging and left against medical advice before being seen by the neurologist.

The patient was continued on her LAI treatment with 25 mg of fluphenazine and remained stable at her baseline during inpatient observation. The long-term prognosis was considered guarded due to chronic psychosis, poor insight, unreliable historical reporting, and suspected neurocognitive deficits. The patient was converted to voluntary status once stabilized on her LAI and refused further services against medical advice on hospital day three. She returned home with her parents with directions to follow up with her psychiatrist for outpatient. She has since been lost to follow-up, and no new admissions or visits have been documented in the 12 months following her discharge.

## Discussion

This case presents a rare and complex instance of chronic psychosis and neuropsychiatric dysfunction, potentially secondary to HIBI due to PPH. While PPP typically manifests acutely and resolves within weeks to months, this patient’s presentation deviated significantly from this expected trajectory, suggesting an organic etiology of her symptoms [2,3]. The chronic disease course, cognitive distortions, and fixed delusions observed in this patient raise important questions regarding the neuropathological sequelae of severe obstetric complications and their role in persistent psychiatric illness [12,13].

Cerebral hypoperfusion following severe PPH is an underrecognized contributor to long-term neuropsychiatric outcomes. Cerebral hypoperfusion during obstetric emergencies may result in diffuse neuronal injury, particularly in vulnerable cortical regions such as the hippocampus, prefrontal cortex, and basal ganglia, which are integral to mood regulation, memory, and executive function [8]. In this case, CT imaging revealed global cortical atrophy significantly out of proportion to her age (Figure 2), suggesting an organic cause for her presentation [14]. MRI is required to accurately characterize anatomical brain changes in the setting of HIBI, but in the absence of MRI imaging, generalized findings on CT offer insight into the organic origins of psychiatric symptomology. Although neurocognitive testing did not reveal any gross abnormalities, her long-term memory deficits, confabulation, and poor insight were consistent with cortical and subcortical dysfunction, as theorized from imaging findings that may not be readily detected using standardized screening tools [4,10-14].

Psychosis following anoxic brain injury has been described in the literature, albeit infrequently, and may involve pathophysiological mechanisms such as neuroinflammation, dysregulation of dopaminergic pathways, and loss of cortical inhibitory control [15,16]. Furthermore, this case illustrates an unusual blend of psychiatric symptoms, including chronic delusions, dissociation, amnesia, and affective flattening characteristics that straddle both psychotic and organic cognitive syndromes [15-17]. This mixed presentation complicates the diagnosis and challenges the clear delineation between primary psychiatric disorders and secondary psychosis due to structural brain abnormality [15-17].

The involvement of obstetrics, psychiatry, and neurology is essential in such cases, not only for accurate diagnosis but also for developing sustainable treatment plans [1,7,11]. Neuroimaging, although underutilized in routine psychiatric evaluations, plays a pivotal role in identifying cortical abnormalities that may otherwise be attributed solely to a primary psychotic process [18,19]. Moreover, this case underscores the limitations of conventional psychiatric instruments in capturing nuanced cognitive and functional impairments in medically complex patients [11,15-17]. It is pertinent to assess other causes of psychosis in the setting of PPH, such as endocrine dysregulation and hypopituitarism [8,9,20]. Although there were no findings to suggest conditions such as Sheehan’s syndrome in this patient, it is imperative that clinicians pursue a thorough medical examination to identify electrolyte abnormalities, endocrine dysfunction, and metabolic syndrome in high-risk patients [8,9,20].

In the absence of definitive biomarkers, longitudinal and functional imaging may be warranted to better understand the interplay between obstetric trauma, hypoperfusion injury, and chronic psychiatric syndromes [7,18]. Early identification and intervention in patients with obstetric complications can mitigate the risk of prolonged psychiatric morbidity and improve long-term outcomes [7].

This case is limited by the lack of available medical records and an incomplete timeline of events. Additionally, the stigmatization of psychiatric illness and mental health evaluation impeded a comprehensive and thoughtful approach to the patient’s care. The history presented here suggests a clear onset of neuropsychiatric symptoms following PPH, indicating a complex underlying pathophysiologic mechanism. We believe that the prior diagnoses of bipolar I disorder and schizophrenia likely represented misdiagnoses stemming from insufficient historical context. A thorough understanding of the sequence of events preceding and following personality or mental status changes after a traumatic event is imperative, yet becomes increasingly challenging as time elapses.

## Conclusions

This case highlights a rare and under-recognized trajectory of chronic psychosis potentially linked to PPH and suspected HIBI due to cerebral hypoperfusion. Unlike the typical course of PPP, this patient demonstrated persistent, treatment-resistant delusions, memory distortion, and impaired insight, compounded by radiographic evidence of global cortical atrophy. Her presentation underscores the importance of considering secondary neuropsychiatric syndromes in the differential diagnosis of chronic psychosis, particularly in patients with a history of severe obstetric complications and hemorrhage.

This case emphasizes the necessity of interdisciplinary collaboration between psychiatry, neurology, and obstetrics in patients with emerging neuropsychiatric symptoms following a PPH. Furthermore, this case highlights the diagnostic value of neuroimaging in complex psychiatric presentations where historical information may be unreliable or unobtainable. Further research is needed to elucidate the long-term neuropsychiatric consequences of hypoxic brain injury following extensive PPH to better inform diagnostic frameworks and therapeutic strategies.
